# The Immunomodulatory Effects of Mesenchymal Stem Cells in Prevention or Treatment of Excessive Scars

**DOI:** 10.1155/2016/6937976

**Published:** 2015-12-29

**Authors:** Bommie Florence Seo, Sung-No Jung

**Affiliations:** Department of Plastic and Reconstructive Surgery, College of Medicine, The Catholic University of Korea, Seoul 06591, Republic of Korea

## Abstract

Excessive scars, including keloids and hypertrophic scars, result from aberrations in the process of physiologic wound healing. An exaggerated inflammatory process is one of the main pathophysiological contributors. Scars may cause pain, and pruritis, limit joint mobility, and cause a range of cosmetic deformities that affect the patient's quality of life. Extensive research has been done on hypertrophic scar and keloid formation that has resulted in the plethora of treatment and prevention methods practiced today. Mesenchymal stem cells, among their multifunctional roles, are known regulators of inflammation and have been receiving attention as a major candidate for cell therapy to treat or prevent excessive scars. This paper extensively reviews the body of research examining the mechanism and potential of stem cell therapy in the treatment of excessive scars.

## 1. Introduction

Excessive scarring, first described in the Smith papyrus about 1700 BC, is a persisting phenomenon that provides a spectrum of morbidities on the inflicted [1]. Specific to humans, they may occur after any type of injury including burns, lacerations, abrasions, piercings, surgical incisions, or injections. Hypertrophic scars or keloids are scars that present with an overabundance of dermal collagen, rising above skin level. Such lesions not only are cosmetically unattractive, but may also limit joint function and cause uncomfortable symptoms such as pain and pruritis. The resulting psychological burden affects the patient's quality of life and escalates health care costs [2].

Although the definitive process underlying such scar formation is yet to be elucidated, the upregulated, exaggerated inflammatory response has been found to be a critical step in achieving excessive scars [3–5]. Normal physiologic wound healing in human adults undergoes three overlapping phases: inflammation, proliferation, and remodeling [6]. Immediately after injury, platelet degranulation and activation of complement and coagulation cascades result in formation of a fibrin clot at the site of injury. This structure provides hemostasis and functions as the seat of wound chemotaxis. This temporary extracellular matrix (ECM) stimulates the recruitment of inflammatory cells (neutrophils, macrophages, epithelial cells, mast cells, endothelial cells, and fibroblasts), which in turn produce proinflammatory mediators including macrophage inflammatory protein-1alpha (MIP-1*α*), monocyte chemotactic protein-1 (MCP-1), RANTES, interleukin-1beta (IL-1*β*), and interleukin-6 [7, 8]. Inflammatory cells also deliver a wide range of growth factors, transforming growth factor-beta 1 (TGF-*β*1), transforming growth factor-alpha (TGF-*α*), basic fibroblast growth factor (bFGF), vascular endothelial growth factor (VEGF), and platelet derived growth factor (PDGF) [9, 10]. Proliferation begins within 48 hours to 10 days after injury, characterized by replacement of the temporary fibrin scaffold with a vascularized ECM synthesized by recruited fibroblasts. The construction of this granulation tissue, composed of procollagen, elastin, proteoglycans, and hyaluronic acid, provides the framework for vascular ingrowth and migration and proliferation of keratinocytes [11, 12]. During this stage, myofibroblasts, modified fibroblasts containing actin filaments, mediate wound contraction to bring wound margins together [4]. With wound closure comes the remodeling phase, beginning around 14 to 21 days after injury. ECM is reorganized and degraded, during which a variety of proteolytic enzymes including matrix metalloproteinases (MMPs), and their inhibitors (TIMPs for tissue inhibitor of MMPs) play a major role. The proportion of type I collagen to type III collagen increases [3]. Maturation of the scar results in a decrease in cellularity and vascularity of the tissue [6]. The number of myofibroblasts is dramatically reduced through the process of apoptosis [13].

It is evident that the transformation of a fibrin clot into mature scar tissue requires a delicate balance between ECM deposition and degradation. Physiological wound healing requires timely resolution of the inflammatory response, and when this process is disrupted abnormalities in scarring will occur. There is substantial evidence in the literature that increased inflammation is a prerequisite for scarring [9, 14]. Fetal mammalian wound healing is scarless, incorporating fewer inflammatory cells and fewer inflammatory mediators, with a shorter inflammatory phase [15]. Oral mucosal wound healing results in reduced scar formation compared to skin, and studies have found that mucosa ECM components resemble that of fetal skin and have reduced inflammatory cell infiltration and proinflammatory mediators [16, 17].

Mesenchymal stem cells (MSCs) are characterized by their regenerative capacity and have been recognized as a legitimate player accelerating the wound healing process [18–21]. MSCs are able to be home to sites of injury, transdifferentiate into epidermal or dermal lineages, and have immunomodulatory, antifibrotic, and angiogenic abilities they exert by secreting an enormous array of paracrine growth factors or cell-to-cell contact [22–25]. Administration of MSCs regulates excessive inflammation, demonstrated by their therapeutic abilities in experimental models of inflammation related fibrotic diseases: lung injury, spinal cord injury, myocardial infarction, corneal injury, renal fibrosis, or liver cirrhosis [24, 26–31]. The ability and mechanisms with which MSCs attenuate the inflammatory process during cutaneous wound healing are an emerging focus of interest, which we will review while overviewing the current tide of research.

## 2. Overview of Current Research

There is a multitude of research that has been and is being performed on the effects of stem cells on the wound healing process. Most experimental designs are composed of stem cells of different origins applied in differing doses via (1) systemic injection, (2) local injection (at the wound site), or (3) seeded on a tissue engineered scaffold at various time points.

### 2.1. Preparation of Stem Cells

The most common type of stem cell used in wound healing and scar research is MSC. Although there have been some studies that applied murine embryonic stem cells (ESCs) to burn wound surfaces, ethical and legislative issues limit the expansion of further investigation [32–34]. MSCs, obtained from human bone marrow aspirate in early studies, are now harvested from various tissues throughout the body [35]. Adipose derived stem cells may be obtained during excisional surgery or liposuction [36]. The marrow space of long bones, periosteum, synovial fluid, nasal septum, gingiva, periodontal ligament, palatine tonsil, parathyroid gland, and fallopian tube also withhold MSCs [37–44]. While adult MSCs are most commonly utilized in research, limitations such as donor morbidity and limitation in proliferative capacity have led researchers to search for an alternative source. Extra-embryonic MSCs harvested after birth from prenatal tissue such as the placenta, umbilical cord, umbilical cord blood/Wharton's Jelly, or the dental pulp of the primary tooth are now major sources of MSCs [45–49].

MSCs in all experimental studies should meet the International Society for Cellular Therapy (ISCT) minimum criteria for MSCs: plastic-adherent when maintained in standard culture conditions, expression of CD105, CD73, and CD90 while lacking expression of CD45, CD23, CD14, or CD11b, CD79alpha or CD19 and HLA-DR surface molecules, differentiation to osteoblasts, adipocytes, and chondroblasts in vitro [50].

### 2.2. Wound Models

It is well known that there is a lack of a universal model of abnormal wound healing [51]. The porcine burn model comes closest in resemblance of human scar tissue; however, most studies were performed on previously established wound models of smaller animals [52]. An excisional or incisional wound is created, or a cytotoxic agent is injected into the dorsal skin of mice, rats, rabbits, or, rarely, pigs [36, 53–59]. Because loose skinned animals, including mice or rats, display rapid wound healing mediated by wound contracture, some studies used splints to resist against this process [58, 60, 61]. Fu and Li and Yun et al. used a burn wound produced by a heated brass bar pressed on the backs of minipigs or male Yorkshire pigs, respectively [62, 63].

### 2.3. Delivery of MSCs

While most earlier studies delivered MSCs to the site of injury via systemic intravenous or local subcutaneous or dermal injection, recent studies have been focusing on a more effective method of transport. Tissue engineering approaches combine stem cells with biomaterial scaffolds or matrices attempt to minimize unprogrammed cell death and/or migration from the wound. The incorporation of scaffolds is supported by researchers who argue that recapitulation of the stem cell microenvironment is necessary to enhance their potential [64, 65]. Acellular dermal matrices carrying adipose-derived stem cells have been effective in targeted cell delivery [66, 67]. Another group has found that poly(hydroxybutyrate-co-hydroxyvalerate) (PHBV), a natural polymer that has previously shown influence on reepithelialization, synergistically acts to downregulate the inflammation process in murine skin wound models [68]. Lam et al. found increased cell survival and proliferation and reduced scarring when they applied a patch harvested from porcine small intestine submucosa (SIS) seeded with adipose-derived stromal cells onto murine skin excision wounds [69]. Fat grafting or cell-assisted lipotransfer into or beneath scar has been suggested as a natural method of delivering autogenous adipose derived stem cells but requires further evidence [70, 71].

The advantages in using tissue engineering include the possibility of designing a 3-dimensional structure tailored to the wound and the potential of attaching biomaterials that are synergistic with the cells.

### 2.4. Clinical Studies

The transition of MSC application from bench to bedside has yet many barriers to overcome. These include issues of safety, efficacy, and cost-effectiveness, among others. More clinical studies are warranted before development of a cell treatment. Most of the research on MSCs and scars involves animal models and is focused on how the cells exert their immunomodulatory abilities during the early stages of the wound healing process. There have been few reports on the use of stem cells in human scars. In 2014, Hemphill et al. found that injection of a heterogeneous mixture of 2 million human amniotic stem cells and amniotic membrane matrix directly into an intractably painful postsurgical scar resulted in a significant decrease in pain and visible decrease in scar tissue [72]. The application of fat grafts into neuropathic scars or hypertrophic scars shows pain alleviation or an increased scar quality. Fat grafts are a source of MSCs and adipose derived stem cells which are thought to be involved in this process [73].

## 3. Immunomodulatory Effects of MSCs That Downregulate Excessive Scarring

### 3.1. MSCs Are Capable of Homing the Site of Injury

MSCs that are injected systemically travel through the circulatory system to ultimately concentrate at the site of tissue damage [24, 74]. MSCs express chemotaxis toward a variety of wound healing cytokines in vitro such as PDGF, TNF-*α*, insulin-like growth factor-1, and IL-8, which explains their preferential migration toward wounds [75, 76].

### 3.2. MSCs Modulate Inflammatory Cells

MSCs produce a vast spectrum of paracrine factors. The main paracrine factors involved in immunomodulation are TGF-*β*, prostaglandin E2 (PGE2), hepatocyte growth factor (HGF), IL-10, IL-6, indoleamine 2,3-dioxygenase (IDO), nitric oxide (NO), and human leukocyte antigen G (HLA-G) [77–79]. Each of these factors is known to regulate different target immune cells. Other than such soluble factors, MSCs secrete extracellular vesicles (EVs), lipid bilayers that contain and transport the cytoplasmic components of the MSCs [25]. EV is an inclusive term that has recently been suggested to encompass both exosomes and microvesicles [80]. Several studies have reported the immunological potential of MSC EVs in vitro, and the ability of these EVs to attenuate an activated immune system in vivo [81, 82]. Along with cell-to-cell (MSC) contact, the MSC secretome including EVs and soluble factors modulates the inflammatory response.

#### 3.2.1. Natural Killer (NK) Cells

MSCs are capable of inhibiting proliferation and function of NK cells, mediated by IDO, PGE2, and TGF-*β*1 [78, 79, 83]. Numerous studies have shown that MSCs only partially inhibit the proliferation of activated NK cells and are susceptible to lysis by activated cells [77]. HLA-G5 inhibits NK cell mediated cytolysis and decreases interferon-gamma (IFN-*γ*) secretion [84].

#### 3.2.2. Dendritic Cells (DCs)

Dendritic cells are antigen presenting cells that differentiate from monocytes or CD34+ hematopoietic stem cells until contact with antigens, after which they are activated into mature cells. MSCs are known to impair this differentiation process via PGE2 secretion [83].

#### 3.2.3. Neutrophils

Neutrophils arrive at the wound through chemotaxis, traversing postcapillary venules to degrade pathogens with the granules within phagolysosomes, and then undergo apoptosis. IL-10 secreted by MSCs inhibit neutrophil invasion into the wound. MSCs secrete TNF-stimulated gene/protein-6 (TSG-6), which interacts with protein ligands to inhibit rolling and transendothelial migration of neutrophils. Dyer et al. have found that TSG-6 interacts with the glycosaminoglycan binding site of CXCL8 (IL-8), a chemokine produced by macrophages and transported to the surface of the endothelium, impairing neutrophil adhesion and migration [85].

#### 3.2.4. Macrophages

Macrophages, early responders that arrive at the injury site hours later than neutrophils, are phagocytes that cleanse the wound of matrix and cell debris. They may be polarized depending on environment and may be typically classified into two main groups: classically activated macrophages (M1) and alternatively activated macrophages (M2). M1 macrophages generally withhold antimicrobial characteristics and promote a Th1 type response while M2 macrophages promote Th2 type responses. The M2 macrophage can be classified into M2a, M2b, and M2c macrophages that are defined by specific patterns of cytokine production. In general, M2 macrophages secrete less proinflammatory cytokines, have high production of anti-inflammatory cytokines such as IL-10, and induce resolution of the inflammatory phase. This is in the exception of M2b macrophages, which maintain high levels of inflammatory cytokines [86]. Many studies have demonstrated the ability of autologous or allogeneic MSCs to polarize macrophages toward an M2 phenotype in vitro mediated by paracrine mechanisms, enhancing expression of M2 associated macrophage genes [87]. Kim and Hematti have suggested a separate definition for MSC-educated macrophages that secrete high IL-10 and IL-6 and low IL-12 and TNF-*α*, to call them M2m, different from other subcategories. They propose the possibility of collecting monocytes through leukapheresis and coculturing these mononuclear cells with allogeneic MSCs to provide MSC-educated macrophages prepared for repair of wounds [87].

#### 3.2.5. B Cells

B lymphocytes are manufacturers of antibodies in response to antigens. MSCs may block B cell proliferation in the G0/G1 phase of the cell cycle without eliciting apoptosis [88]. Krampera et al. found that inhibition of proliferation was seen only in the presence of IFN-*γ*, which is probably mediated by MSC production of IDO. IDO is the first and rate-limiting enzyme of the essential amino acid tryptophan catabolism to kynurenine pathway, causing depletion and therefore halting growth. IFN-*γ* has IDO inducing effects [89]. The differentiation of B cells is also inhibited in the presence of B cells [88].

#### 3.2.6. T Cells

Inhibitory effects of T cell proliferation by MSCs are mediated by both cell-to-cell contact and soluble factors. TGF-*β*1 and HGF work together to suppress T cell proliferation [90]. MSCs secrete PGE2 which prevents differentiation of CD4+ T cells into Th17 cells. MSCs also release IDO and enhance secretion of IL-10, which also inhibit cell proliferation [78]. NO has also been implicated as a mediator that downregulates T cell proliferation by phosphorylation of signal transducer and activator of transcription-5 (STAT5). STAT5 is a transcription factor required in activation and proliferation of T cells [91].

### 3.3. MSCs Downregulate Fibrosis

The major downregulators of fibrosis produced by MSCs are PGE2, IL-10, NO, HGF, and adrenomedullin. When cocultured with T cells, MSCs have increased expression of PGE2. PGE2 is produced from arachidonic acid with the aid of enzymes cyclooxygenase-1 or cyclooxygenase-2 [79]. PGE2, already described to inhibit or reduce proliferation or function of NK cells, DCs, T cells, and Treg cells, also induce T cells and macrophages to express higher levels of IL-10 [92, 93]. IL-10 is a major anti-inflammatory cytokine that inhibits neutrophil infiltration into the wound. Neutrophils are one of the first-responders to the inflammatory reaction, capable of ingesting microorganisms, releasing granules filled with enzymes and antimicrobial proteins, and constructing neutrophil extracellular traps that trap and kill microbes extracellularly. During phagocytosis, there is a burst of oxygen consumption, and much of the extra oxygen consumed is converted to highly reactive oxygen species (ROS) (“respiratory burst”) [94]. Therefore IL-10 works to prevent further damage from neutrophil release of ROS. ROS are cytotoxic and have antimicrobial effects but also damage normal tissue and intensify collagen deposition by causing membrane lipid oxidation and induction of TGF-*β* [95]. NO are known to scavenge ROS, resulting in reactive nitrogen species which are less toxic.

HGF is a growth factor secreted by MSCs that modulate fibroblasts, the central player in fibrosis. Myofibroblasts, rich in alpha smooth muscle actin (SMA-*α*), are responsible for wound contraction and secretion of ECM and undergo apoptosis after wound maturation. The continued presence and activation of myofibroblasts is seen during excessive scarring. HGF downregulates fibroblast expression of TGF-*β*1, which drives myofibroblast differentiation, and collagens types I and III [96]. HGF upregulates fibroblast expression of MMPs, therefore enhancing degradation of the ECM. HGF also acts on keratinocytes, upregulating expression of VEGF-A, and is shown to induce angiogenesis without vascular inflammation [97, 98].

### 3.4. MSCs Are Able to Differentiate and Transdifferentiate into Dermal or Epidermal Cell Types

MSCs are characterized by their ability to differentiate and transdifferentiate into cells of different lineages. Capability to differentiate into osteoblasts, adipocytes, and chondrocytes in vitro is included in the criterion of MSCs. However, when cocultured in vitro with keratinocytes, MSCs show transdifferentiation to keratinocytes [99, 100]. These results suggest that MSCs themselves may participate in regeneration of wound tissue.

### 3.5. MSCs Promote Angiogenesis

**Figure 1:**
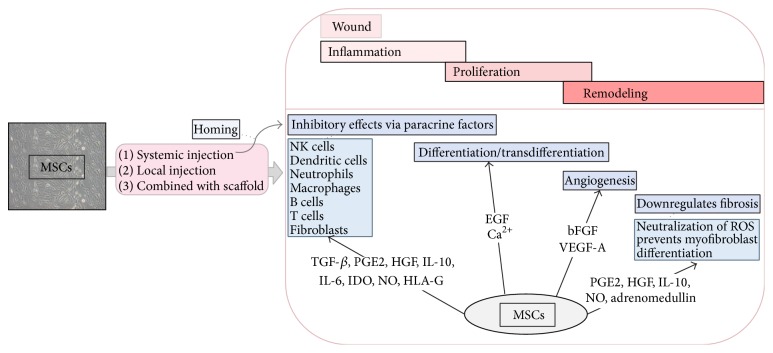
A summary of the immunomodulatory effects of MSCs that downregulate excessive scarring. MSCs are able to home the wound, where the stages of wound healing (inflammation, proliferation, and remodeling) are in progress. MSCs have been found to attenuate the activity of inflammatory cells, differentiate or transdifferentiate into epidermal cell lineages, escalate angiogenesis, and decrease fibrosis.

MSCs are recognized as powerful producers of bFGF and VEGF-A, growth factors that promote proliferation, migration, and differentiation of endothelial cells. Angiogenesis with stable vessels aids the normal progression of wound healing [101]. A summary of the immunomodulatory effects of MSCs can be seen in Figure 1.

## 4. Proinflammatory Capabilities of MSCs

Although the immunomodulatory functions of MSCs have been extensively investigated, there are also reports of proinflammatory capacities of these stem cells. This paradoxical ability has been noted under stimulation of certain infectious molecules. MSCs can be polarized into two uniform but distinct populations, MSC1 and MSC2 [102]. MSC1 is known to express proinflammatory factors while MSC2 expresses immunosuppressive factors. Toll-like receptors (TLRs), a family of transmembrane, immune regulatory pattern recognition receptors, play an important role in MSC-mediated immune responses. Thirteen analogs have been identified, and TLRs 1 through 6 are expressed at higher levels in human and murine MSCs. TLR3 and TLR4 agonists have been found to decrease the ability of MSCs in suppressing T cell proliferation [103]. Blockage of the TLR4 pathway is reported to lead to decrease in B cell activating factor, BAFF, a vital survival factor of B lymphocytes [104].

More clarification is required in the investigation of such proinflammatory pathways. Whether these findings exert an influence at the clinical level remains to be elucidated.

## 5. Conclusion

While there is an escalating volume of preclinical studies on the effect of MSCs on excessive scarring, more long term clinical studies are required to enable translation of this potential into treatment modalities. Issues such as cost effective methods of stem cell harvest, quantification, and delivery must also be addressed. Most research is focused on the prophylactic application of MSCs, applied during the wound healing process before scar maturation. The lack of a universal hypertrophic scar or keloid model may be partially responsible. However, many of patients tend to seek management for scars long after the remodeling phase of wound healing has finalized. Research on the effect of MSCs as a treatment modality of excessive scars may provide a promising solution for many other affected patients. Finally, although MSCs withhold enormous capacities for stem cell therapy in scar management, further insight into polarization and proinflammatory capacities is required before safe application in clinical treatment.
